# Initiation of Early Osteoblast Differentiation Events through the Direct Transcriptional Regulation of Msx2 by FOXC1

**DOI:** 10.1371/journal.pone.0049095

**Published:** 2012-11-07

**Authors:** Farideh Mirzayans, Rotem Lavy, Jonathan Penner-Chea, Fred B. Berry

**Affiliations:** 1 Department of Medical Genetics, University of Alberta, Edmonton, Alberta, Canada; 2 Department of Surgery, University of Alberta, Edmonton, Alberta, Canada; Feinberg Cardiovascular Research Institute, Northwestern University, United States of America

## Abstract

Hierarchal transcriptional regulatory networks function to control the correct spatiotemporal patterning of the mammalian skeletal system. One such factor, the forkhead box transcription factor FOXC1 is necessary for the correct formation of the axial and craniofacial skeleton. Previous studies have demonstrated that the frontal and parietal bones of the skull fail to develop in mice deficient for Foxc1. Furthermore expression of the Msx2 homeobox gene, an essential regulator of calvarial bone development is absent in the skull mesenchymal progenitors of Foxc1 mutant mice. Thus we sought to determine whether Msx2 was a direct target of FOXC1 transcriptional regulation. Here, we demonstrate that elevated expression of FOXC1 can increase endogenous Msx2 mRNA levels. Chromatin immunoprecipitation experiments reveal that FOXC1 occupies a conserved element in the *MSX2* promoter. Using a luciferase reporter assay, we demonstrate that FOXC1 can stimulate the activity of the both human and mouse *MSX2* promoters. We also report that reducing *FOXC1* levels by RNA interference leads to a decrease in *MSX2* expression. Finally, we demonstrate that heterologous expression of *Foxc1* in C2C12 cells results in elevated alkaline phosphatase activity and increased expression of Runx2 and Msx2. These data indicate that *Foxc1* expression leads to a similar enhanced osteogenic differentiation phenotype as observed with *Msx2* overexpression. Together these findings suggest that a *Foxc1*->*Msx2* regulatory network functions in the initial stages of osteoblast differentiation.

## Introduction

The development of the skeleton can proceed via two distinct mechanisms: endochondral and intramembranous ossification [1], [2]. Endochondral ossification involves the prior establishment of a cartilaginous template, formed from osteochondral mesenchyme progenitor cells differentiating into chondrocytes, that is subsequently replaced by bone-forming osteoblasts. Intramembranous ossification involves the direct differentiation of osteochondral mesenchyme progenitor cells into bone-forming osteoblasts without a cartilage intermediate. The long bones of the limbs and other components of the appendicular skeletal system arise through endochondral ossification, whereas bones in the skull arise through intramembranous ossification events.

The regulation of bone formation events needed for correct growth and patterning of the skeletal system is controlled by a network of transcriptional regulatory proteins [3]. The forkhead box transcription factor FOXC1 is required for normal development and patterning of bones originating from both endochondral and intramembranous origins [4], [5], [6], [7]. Targeted deletion of the *Foxc1* gene in mice results in numerous defects in the axial skeleton. The dorsal neural arches of the vertebrae do not ossify and the lateral arches and vertebral bodies are reduced in size. [6]. The rib cage and sternum also display severe ossification defects in the *Foxc1* homozygous null embryos with many fused, misshapen and fragile ribs [5], [6]. The craniofacial skeleton is also severely affected in *Foxc1* ^−/−^ mutant mice, as there is a complete absence of the skull vault. The bones that form the skull vault, or calvarium, are comprised of the frontal, parietal and intraparietal bones. In *Foxc1* null mice only rudimentary calvarial bones are observed near the sites of initial mesenchyme cell condensations [6], [7]. In addition to the skull vault phenotypes, patterning defects to the base of the skull, the basiooccipital bone, and to the hyoid bones are observed in Foxc1 mutant mice [6]. Furthermore, expression of two genes critical in the formation of the mouse craniofacial skeleton, *Alx4* and *Msx2*, are reduced in *Foxc1* mutant mice [7], [8], [9]. The above data indicate an important role for FOXC1 in the formation of the axial and craniofacial skeleton. However, how FOXC1 functions in these processes is not entirely known.

The MSX2 transcription factor is also a critical regulator required for bone formation and development of the craniofacial skeleton. In humans, gain of function mutations in the *MSX2* genes results in Craniostynostosis, Boston Type, a premature fusion of the cranial sutures [9]. In contrast, *MSX2* loss of function mutations cause delays in the formation of cranial sutures [10], [11]. *Msx2*-deficient mice exhibit defective proliferation of osteoprogenitors in the developing calvaria, have defects of skull ossification and persistent interparietal foramina, all of which are reminiscent of the phenotype in humans [12]. As described above, levels of Msx2 expression are greatly reduced in the developing skull vault of *Foxc1* mutant mice, suggesting this gene is under direct regulation by Foxc1 [7]. In this report we demonstrate that expression of *Msx2* is directly regulated by FOXC1 through the binding of a conserved FOXC1 consensus regulatory element in the promoters of the mouse and human Msx2 genes. Furthermore, we demonstrate that heterologous expression of *Foxc1* in C2C12 cells results in a similar enhanced osteogenic differentiation phenotype to that observed with *Msx2* overexpression. Together these findings suggest that a *Foxc1*->*Msx2* regulatory network functions in early stages of osteoblast differentiation.

## Materials and Methods

### Plasmids

*FOXC1* expression plasmids have been described previously [13], [14]. The human and mouse *MSX2* promoters were amplified from genomic DNA using the following primers: Human *MSX2* forward 5′-gctagcgaacttattctggcggtagagg-3′; Human *MSX2* reverse 5′-aaggcttcatgacttctctgccctagc-3′; Mouse *Msx2* forward 5′-gctagcgcagatttccaacattctcagg-3′; Mouse *Msx2* reverse 5′-agatcttccgacgaaaacaagtcacc-3′. DNA fragments were cloned into the NheI and HindIII (human) or BglII (mouse) sites of pGL3-Basic. The vector pBABE-FOXC1 was created by inserting the full length human *FOXC1* cDNA into the EcoRI and SalI sites of pBABEpuro.

### Cell Culture

U2OS, CH310T1/2 (herein referred to as 10T1/2, MDA MB231, HEK293T and C2C12 cells (obtained from ATCC) were cultured in Dulbecco’s Modified Eagle Media (DMEM) supplemented with 10% Fetal Bovine Serum (FBS). For transient transfections cells were plated 24 hours before transfection at a concentration of 4×10^4^ cells per ml. The next day, cells were transfected with cDNA expression vectors using FuGENE6 reagent. We typically transfect cells with a ratio of 3 µl of FuGENE6 per 1 µg DNA. Forty-eight hours after transfection cells were harvested for protein, RNA, or luciferase assays.

### RNA Isolation and q-RT PCR

RNA was isolated from cells using the RNAeasy Mini Kit as described by the manufacturer (Qiagen). Five hundred nanograms of RNA were used in reverse transcription reactions. RT reactions were subsequently diluted 1∶50 and used for quantitative RT PCR reactions. Kapa SYBR-FAST qPCR kits were used as per the manufacturer’s protocols to detect changes in gene expression using the ΔΔCT method. Samples were run in triplicate on an ABI Prism 7900HT thrermocycler or BIO-RAD CFX96 Touch real time PCR detection system. Primers for qRT-PCR were selected from the Primer bank database [15]. Statistical analysis of real-time PCR data was determined by Student T-test or Mann-Whitney U test using SigmaPlot version 12.

### Chromatin Immunoprecipitation (ChIP)

ChIP assays were performed as described previously [16]. Briefly, sheared, cross-linked chromatin from 10T1/2 cells was incubated with 1 µg anti-FOXC1 antibody (Abcam) or with normal goat IgG overnight at 4°C. Magnetic Protein-G beads (25 µl) that were blocked with BSA and salmon sperm DNA were added and reactions continued for a further 2 hours. After four washes with ChIP Wash Buffer (20 mM Tris-Cl, pH 8.0; 150 mM NaCl; 2 mM EDTA; 0.1% SDS and 1% Triton X-100) and a single wash in ChIP Final Wash Buffer (20 mM Tris-Cl, pH 8.0; 500 mM NaCl; 2 mM EDTA; 0.1% SDS and 1% Triton X-100), the DNA was eluted from the beads and the crosslinking was reversed. Qiagen PCR cleanup kit was used to isolate ChIP DNA. A region flanking the putative FOXC1 binding site in the mouse Msx2 promoter was isolated with the following primers: 5′-gcagatttccaacattctcagg-3′ (forward) and 5′-gcagagttgttattaggcgatcc-3′ (reverse). As a negative control, primers corresponding to exon 2 of mouse Msx2 gene were used (forward 5′gcacacccttcaccacatcc-3′; reverse 5′-agggaagggcagactgaagc-3′). ChIP products, along with input chromatin fraction (1%), were amplified on a BIORAD CFX96 touch. Ct values for anti-FOXC1 ChIP and mock (IgG) ChIP were analyzed for statistical significance using T-test. Data were presented as percentages of Input DNA amplification signals.

### Luciferase Reporter Assays

Reporter gene assays were performed in 10T1/2 cells. Cells were seeded into 24 well plates 24 hours prior to transfection at a density of 4×10^4^ cells per well. The following day, cells in each well were transfected with 50 ng of human or mouse MSX2 reporter, along with 250 ng of pcFOXC1 or empty pcDNA4 expression vectors and 10 ng of RL-TK. Dual luciferase assays were performed as per the manufacturer’s instructions (Promega). Each experiment was performed in triplicate and all experiments were repeated three times.

### Electrophoretic Mobility Shift Assays (EMSA)

We utilized a non radioactive EMSA to detect FOXC1-DNA interactions using 5′-IR700 labeled oligonucleotides. Protein lysates (25 µg) from U2OS cells transfected with Xpress-tagged FOXC1 or un-transfected cells were incubated with a 4× EMSA binding buffer (40 mM Hepes, 20% glycerol, 100 mM NaCl, 8 mM DTT, 0.4 mM EDTA, 0.2 µg/µl poly dI-dC) for 10 minutes at room temperature. Probe was then added (50 nM final concentration) to each reaction and incubated for 20 minutes at room temperature. A 6% poly-acrylamide Tris-glycine-EDTA gel was pre-run at 105 V for 15 minutes. Samples were then loaded onto the gel, and run at 95 V for 1 hour. The gel was scanned using a Li-Cor Odyssey Infrared Imaging System.

**Figure 1:**
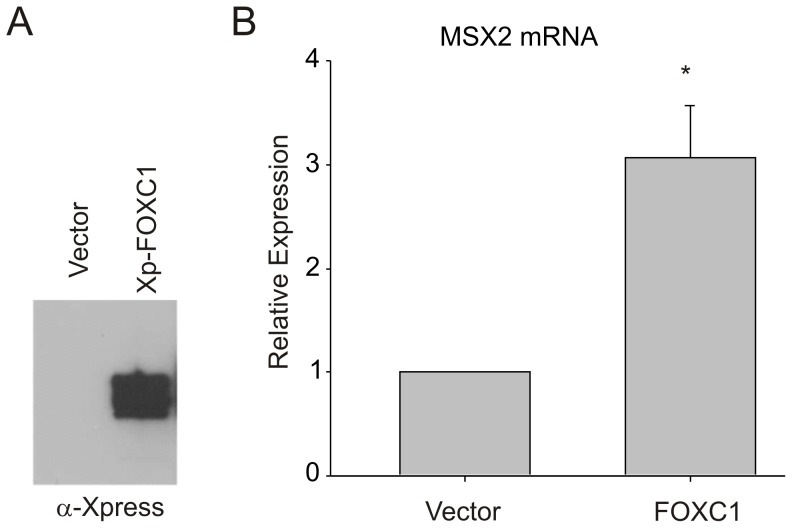
FOXC1 induces *MSX2* expression. (A).U2OS cells were transiently transfected with Xpress-tagged *FOXC1* expression vectors. Levels of exogenous FOXC1 protein were determined by immunoblotting with anti-Xpress antibody. (B) *MSX2* mRNA levels were measured by qRT-PCR in FOXC1-over expressing cells. Error bars represent the standard error of the mean. *, p value <0.0001.

**Figure 2:**
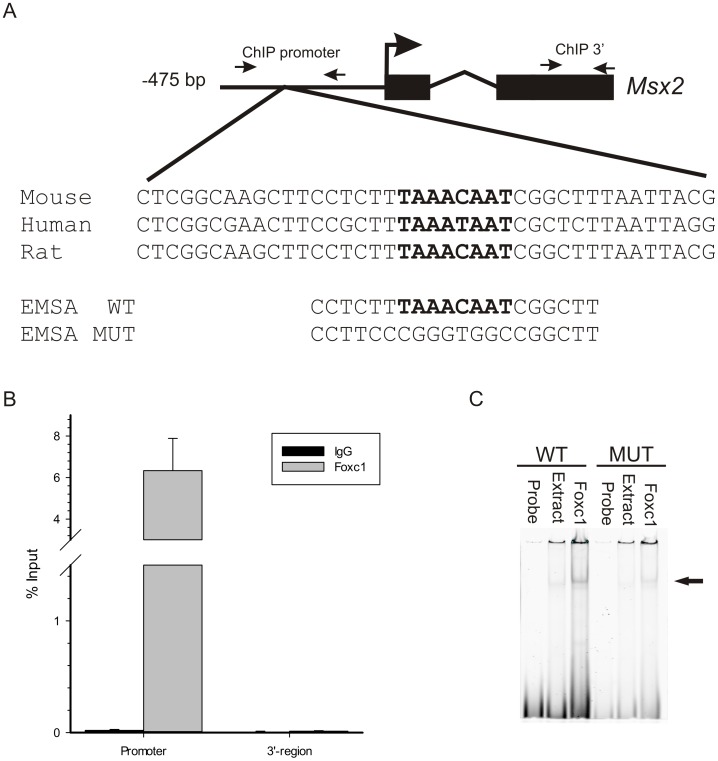
FOXC1 binds to the *Msx2* promoter in vivo. (A) Sequence analysis of upstream regulatory elements reveals the presence of a FOXC1 binding motif, indicated in bold, (TAAAT/CAAT) located in a conserved motif near the predicted *Msx2* transcription start site of the mouse, rat and human genes. Small arrows correspond to the position of ChIP primers located in the promoter region or the coding region of mouse *Msx2*. Nucleotide sequences of the Electrophoretic mobility shift assay (EMSA) probes for wild type (WT) and mutated (MUT) FOXC1 binding sites are indicated. (B) Chromatin immunoprecipitation assays confirm the binding of FOXC1 to the *Msx2* promoter *in vivo*. Quantitative PCR (qPCR) was conducted on ChiP products isolated from 10T1/2 cells using antibodies recognizing FOXC1 or normal immunoglobulins (IgG). Primers were designed amplify regions in the promoter flanking the putative FOXC1 binding site or exon 2 of the mouse Msx2 gene. Amplification signals are presented a percentage compared to input chromatin fraction. (C) EMSAs demonstrate FOXC1 binding to DNA elements in the Msx2 promoter. Extracts from U2OS cells or cells transfected with FOXC1 were incubated with IR700-labeled oligonucleotides correspond to the WT or MUT FOXC1 binding sites. FOXC1-DNA complexes are indicated by the arrow.

### Virus Production and Transduction

Lentiviral vectors (pLKO.1) expressing shRNAs targeting human *FOXC1* were purchased from Open Biosystems. The vector pLKO1-EGFP, which produces shRNAs targeting enhanced green fluorescent protein (EGFP) was used as a negative control. HEK-293T cells (1.5×10^5^ cells) were transfected with psPAX2 (900 ng), pMD2.G and pLKO1-FOXC1 (1 µg) or pLKO1-EGFP (1 µg) in a 60 mm tissue culture plate. Eighteen hours following transfection the media was removed and replaced with 6 ml of high serum (30%) DMEM. Media was collected every 12 hours for 48 hours. Pooled media collections were centrifuged at 500×g to remove any remaining packaging cells. For lentiviral infection, MDA-MB231 cells were subcultured at a density of 5×10^5^ cells in a 60 mm tissue culture plate the day prior to infection. The following day, the media was removed and replaced with 6 ml fresh growth media containing polybrene (8 µg/ml final concentration) and 500 µl of lentiviral supernatant. Twenty-four hours later the media was removed and replaced with fresh growth media containing 0.5 µg/ml puromycin. After 4 days of selection, resistant colonies (<200) were pooled and expanded. For retrovirus production, HEK293T cells were transfected with pBABE (1 µg) or pBABE-FOXC1 (1 µg) along with pCL-ECO (1 µg) as above. Virus containing supernatants were collected as above and filtered through a 0.45 μ filter. C2C12 cells were infected and selected in a fashion similar to MDA-MB231 cells.

**Figure 3:**
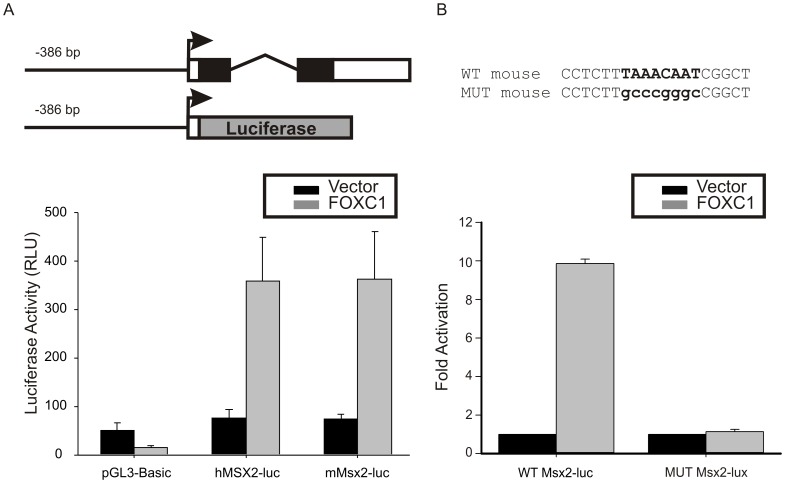
FOXC1 activates the *MSX2* promoter. (A) Luciferase (luc) reporter vectors consisting of the human (h) or mouse (m) *MSX2* promoter which contained the conserved FOXC1 binding element were created. Transfection of 10T1/2 cells with *FOXC1* expression vectors lead to a robust activation of both human and mouse *MSX2* promoters. (B) The putative FOXC1 binding sites was mutated in the mouse Msx2-luc reporter. Wild type and mutated Msx2-luciferase vectors were co-transfected with empty pcDNA4 (vector) or Xpress tagged-FOXC1. Error bars correspond the standard error of the mean.

### Osteogenic Differentiation

Wild type C2C12, C2C12-BABE and C2C12-FOXC1 cells were seeded at a density of 2.5×10^5^ cells per well of a 6 well plate. Four days later, the cells were prepared for RNA isolation or fixed in 4% parformaldehyde and washed with PBS. To detect alkaline phosphatase activity, cells were incubated with BCIP/NBT liquid substrate system overnight.

## Results

### FOXC1 Positively Regulates MSX2 mRNA Expression

*Msx2* mRNA expression is reduced in the mesenchyme progenitor cells that form the skull in *Foxc1* mutant mice, suggesting *Msx2* as a putative target gene for FOXC1 regulation [7]. In order to test whether FOXC1could regulate the expression of endogenous *MSX2*, we over expressed FOXC1 in U2OS human osteosarcoma cells and measured *MSX2* mRNA expression by qRT-PCR. As indicated in Fig. 1, levels of endogenous *MSX2* mRNA were elevated by 3 fold compared to control transfected cells. These data suggest that FOXC1 positively regulates expression of endogenous *MSX2* mRNA.

**Figure 4:**
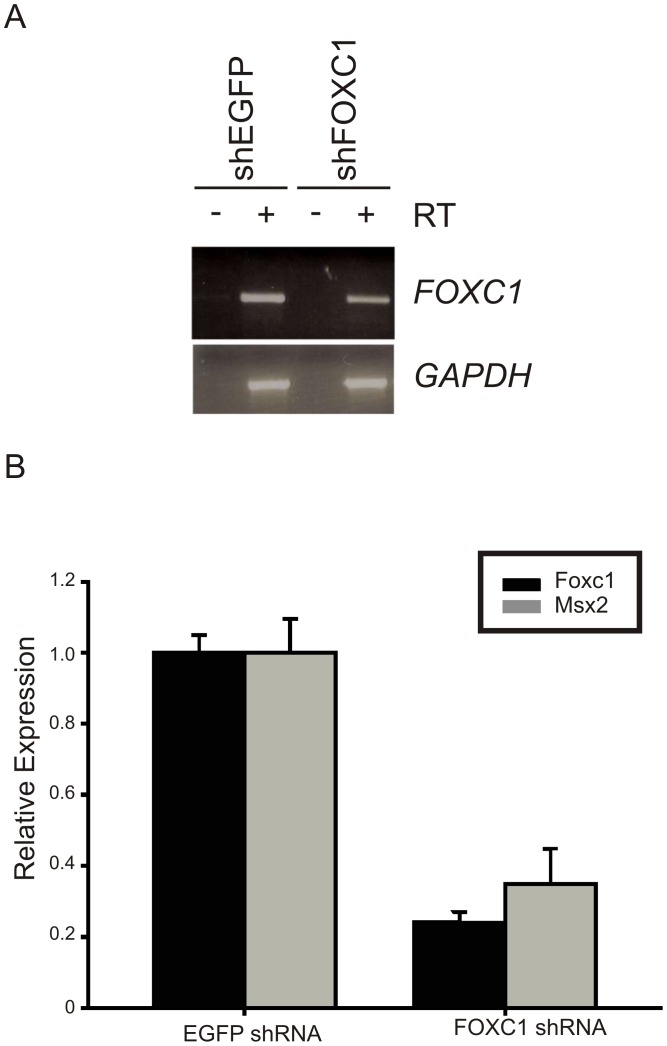
*FOXC1* shRNA expression reduces *MSX2* mRNA levels. (A) MB231 cells were transduced with lentiviral particles containing EGFP or FOXC1 shRNA. Pooled puromycin resistant colonies were expanded and *FOXC1* expression analyzed semiquatitative rt-PCR. (B). qRT-PCR data indicating reduced expression of *MSX2* mRNA in MB231 cells when *FOXC1* levels are reduced by RNA interference.

### FOXC1 Binds to and Activates the MSX2 Promoter

To assess whether *Msx2* is indeed a direct target of FOXC1 transcriptional regulation, the upstream regulatory regions of human and mouse genes were surveyed for the presence of a FOXC1 binding motif using a position weight matrix for the FOXC1 recognition site. FOXC1 recognizes a core 5′-TAAAT/CAA-3′ consensus sequence [14], [17] and such sequence was found approximately 280 bp upstream of the transcription start site in the mouse, human and rat *Msx2* genes (Fig. 2). We designed primers flanking this putative FOXC1 binding site in the mouse gene and performed chromatin immunoprecipitation experiments in 10T1/2 mouse mesenchymal cells. As indicated in Figure 2B, a DNA fragment corresponding to the FOXC1-binding element described above was successfully recovered by ChIP with α-FOXC1 antibodies, indicating that FOXC1 was in fact bound to this region. No amplification signal was detected with primers amplifying the 3′ coding region of the *Msx2* gene. Finally, we performed EMSAs to confirm binding of FOXC1 to *Msx2* regulatory regions. Cell extracts expressing FOXC1 were able to bind an oligonucleotide probe corresponding the putative FOXC1 binding site (Fig. 2C), but not a probe containing a mutated sequence.

**Figure 5:**
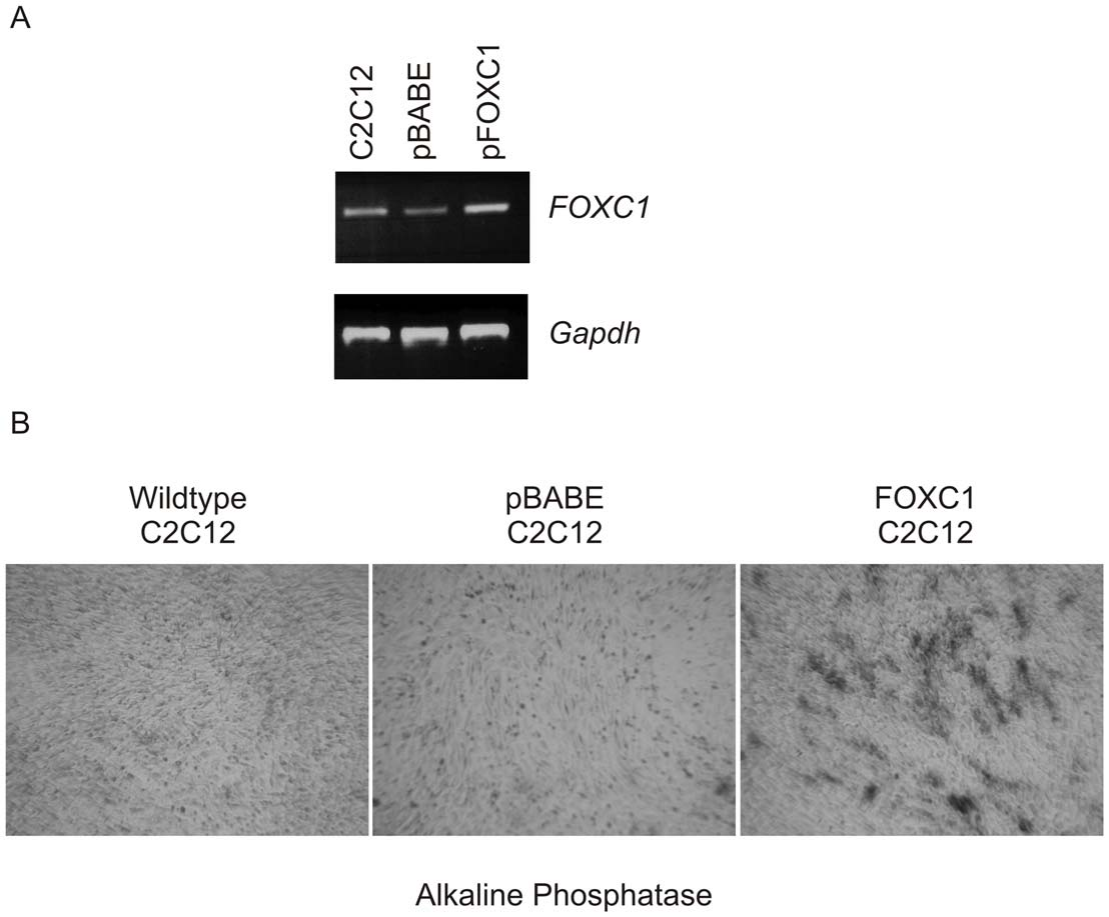
FOXC1 overexpression induced ectopic osteoblast transdifferentiation of C2C12 cells. C2C12 myoblasts were transduced with retroviruses containing pBABE (empty vector control) or pBABE-FOXC1. Cells were grown to 95% confluence and stained for alkaline phosphatase activity after 4 days.

**Figure 6:**
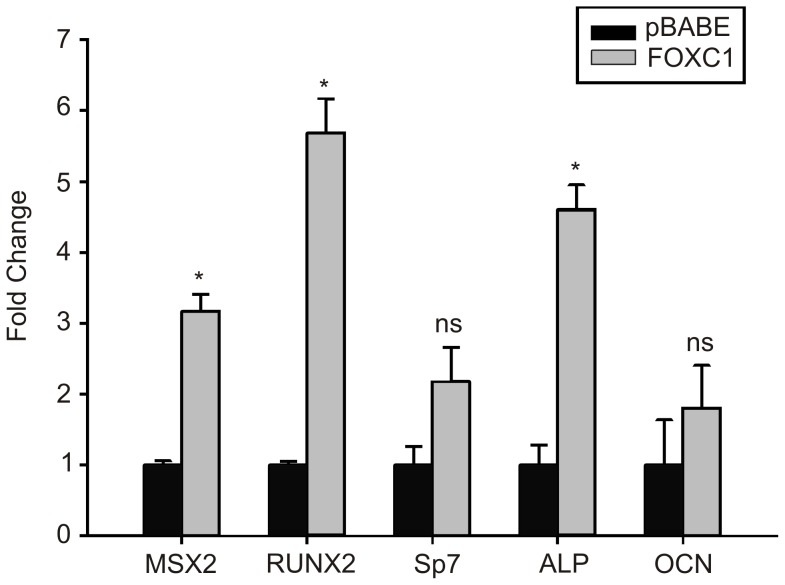
Increased expression of osteogenic marker genes in FOXC1-expressing C2C12 cells. Levels of *Msx2*, *Runx2*, *Sp7* (*Osterix*), Alkaline Phosphatase (Alp) and Osteocalcin (Ocn) mRNA levels were determined by qRT-PCR from C2C12 cells transduced with empty pBABE or FOXC1 retroviral particles. * p<0.05; ns, not significant.

Next we cloned the promoter elements of mouse and human *Msx2* genes into the pGL3 basic luciferase reporter and tested for promoter activity. We created luciferase reporters containing the proximal promoter along with 475 and 482 bp region of the mouse and human *Msx2* genes, respectively, containing the FOXC1 binding site. We tested whether FOXC1 could activate expression from these reporters in CH310T1/2 cells. As indicated in Fig. 3A, robust activation of both the human and mouse reporter construct was observed upon cotransfection of a *FOXC1* cDNA. Together, these data suggest that *Msx2* is indeed a target of FOXC1 transcriptional regulation in mesenchymal cells. We then mutated the FOXC1 binding site in the mouse *Msx2-*luc reporter and observed that FOXC1 was unable to activate this reporter (Fig. 3B).

### Loss of FOXC1 Expression Reduces MSX2 mRNA Levels

Given that *Msx2* expression was markedly reduced in the developing skull of Foxc1 mutant mice [7], we wished to test whether loss of *FOXC1* expression in human cells would reduce levels of endogenous *MSX2* mRNA. *FOXC1* and *MSX2* are expressed in MDA-MB231 human mammary adenosarcoma cells (Fig. 4). MDA-MB231 cells were transduced with lentiviral particles containing shRNAs targeting human *FOXC1* or *EGFP*, as a control. Expression of both *FOXC1* and *MSX2* mRNAs were reduced by over 60% in cells transduced with the *FOXC1* shRNA (Fig. 4B), indicating that, like in *Foxc1* mutant mice, *MSX2* mRNA levels are reduced in response to decreased levels *FOXC1*.

### Activation of the Osteogenic Differentiation Pathway in the Presence of FOXC1 Expression

Overexpression of *Msx2* into C2C12 myoblasts cells results in ectopic osteoblast transdifferentiation of this cell type [18]. To test the biological significance regulation of *Msx2* expression by FOXC1 we created stable C2C12 cells lines constitutively expressing the human *FOXC1* cDNA using retrovirus transduction. When FOXC1-expressing C2C12 cells (FOXC1-C2C12) reach confluence, we observe increased staining of alkaline phosphatase (ALP) activity (an early marker of osteoblast differentiation). No alkaline phosphatase activity is observed in confluent parental C2C12 cells or those transduced with empty pBABE retroviruses (Fig. 5). A similar increase in ALP activity has been observed when C2C12 cells are transduced with *MSX2* expressing viruses [18]. Finally, we monitored the expression of genes that regulate early and mid osteogenic differentiation events. As indicated in Figure 6, levels of *Msx2, Runx2* and *Alkaline phosphatase* mRNA levels are all increased in FOXC1-C2C12 cells compared to pBABE-transduced control cells. No significant changes in mRNA levels were detected for *Osterix (Sp7), Osteocalcin (Ocn)* or *Dlx5* (Figure 6, data not shown). Together these data indicate the elevation of FOXC1 expression can activate expression of osteoblast differentiation regulatory factors.

## Discussion

Networks of transcriptional regulatory proteins are critical for the correct coordination of cellular events that drive formation of the skeleton. Here we describe that the *Msx2* transcription factor gene is a direct target of transcription regulation by the forkhead box transcription factor FOXC1. We demonstrate that FOXC1 elevates endogenous expression levels of *MSX2* mRNA, stimulates activity of a *MSX2* luciferase promoter construct and binds to a conserved FOXC1 binding element in the *Msx2* promoter region. Furthermore, we demonstrate that reducing *FOXC1* expression through shRNA concomitantly reduces *MSX2* mRNA expression. Finally we demonstrate that enforced *FOXC1* expression in C2C12 cells results in the precocious initiation of osteoblast differentiation, similar to what has been observed in with ectopic *Msx2* expression [18]. Together these data indicate that *MSX2* is a *bona fide* target gene of FOXC1 transcriptional regulation.

We have identified a FOXC1 binding element that is conserved in the proximal promoter of mouse, rat and human *Msx2* genes. Using chromatin immunoprecipitation assays, we demonstrate that FOXC1 protein was bound to this region in mouse mesenchymal cells. Furthermore, luciferase reporter gene assays revealed that this promoter region from both human and mouse genes was activated in the presence of Foxc1. A growing list of FOXC1 target genes is beginning to emerge [16], [19], [20], [21], [22], [23], [24]. The identification of such target genes in bone-forming cells will aid in our understanding of how FOXC1 contributes to skeletal growth and patterning.

Both *Foxc1* and *Msx2* are important regulators of skeletal development, especially in the formation of the craniofacial and axial skeleton [6], [7], [9], [10], [11], [12], [25], [26], [27], [28], [29]. In *Foxc1^−/−^* mutant mice, craniofacial skeleton displays rudimentary calvarial bones (frontal, parietal and intra parietal bones) that fail to grow apically [7], [30]. In the developing calvarium, *Foxc1* is expressed in mesenchymal condensations at E11. As development proceeds (E12–E15), expression of *Foxc1* remains elevated in the mesenchyme of the growing calvarial bones and later in the suture mesenchyme, consistent with a role for FOXC1 in early osteoblast differentiation events. Mutations in either *Foxc1* or *Msx2* results in defects in the neural arches of the developing vertebral bones in mice [5], [28]. In humans, FOXC1 mutations result in Axenfeld-Rieger Malformations, an autosomal dominant disorder characterized by craniofacial, ocular and dental anomalies [31], [32]. Given the roles for *Msx2* in craniofacial skeleton and tooth formation [33], [34], we hypothesize that impaired expression of *MSX2* caused by FOXC1 loss of function mutations, may contribute, in part, to the craniofacial and dental phenotypes of Axenfeld Reiger malformations.

We report that FOXC1 is a factor that may function during the initial stages of osteoblast differentiation. FOXC1 expression can be readily detected in mesenchyme condensations prior to the onset of osteogenic differentiation events and expression levels decrease as osteoblast differentiation commences [6], [7], consistent with a role for FOXC1 in early differentiation events. Heterologous expression of *FOXC1* in mouse C2C12 myoblasts resulted in the ectopic transdifferentiation of these cells into osteoblasts with a concomitant increased expression of *Msx2* and *Runx2*, early markers of osteogenesis. We did not observe a significant increase in *Sp7* (*Osterix*) expression or *Dlx5*, two transcription factors that act later in osteogenic differentiation events. Nor did we observe any significant increase in *Osteocalcin* mRNA levels, suggesting FOXC1 function is limited to early events. Similarly, transduction of C2C12 cells with *Msx2* adenovirus will lead to the induction of *Runx2* expression and the initiation of osteogenic differentiation events [18]. The mechanisms in which FOXC1 participates to regulate bone formation are not known. Here we demonstrate that FOXC1 directly regulates expression of *Msx2*, a key regulator of early osteogenic events. Treatment of C2C12 myoblasts with bone morphogenetic proteins will alter the differentiation capacity of these cells from myogenic to osteogenic fates [35]. In our studies, heterologous expression of *FOXC1* in these cells is also sufficient to induce transdifferentiation into osteoblasts cells. Thus, our data suggest that FOXC1 may be involved in regulating the initial differentiation events that direct mesenchymal cells to osteoblasts.

## References

[1] Lefebvre V, Bhattaram P (2010) Vertebrate skeletogenesis. Curr Top Dev Biol 90: 291–317. 20691853 10.1016/S0070-2153(10)90008-2 PMC3077680

[2] Karsenty G, Kronenberg HM, Settembre C (2009) Genetic control of bone formation. Annu Rev Cell Dev Biol 25: 629–648. 19575648 10.1146/annurev.cellbio.042308.113308

[3] Karsenty G (2008) Transcriptional control of skeletogenesis. Annu Rev Genomics Hum Genet 9: 183–196. 18767962 10.1146/annurev.genom.9.081307.164437

[4] Gruneberg H (1951) Genetical studies on the skeleton of the mouse. VII. Congenital hydrocephalus. J Genet 45: 327–358.

[5] Hong HK, Lass JH, Chakravarti A (1999) Pleiotropic skeletal and ocular phenotypes of the mouse mutation congenital hydrocephalus (ch/Mf1) arise from a winged helix/forkhead transcriptionfactor gene. Hum Mol Genet 8: 625–637. 10072431 10.1093/hmg/8.4.625

[6] Kume T, Deng KY, Winfrey V, Gould DB, Walter MA, et al (1998) The forkhead/winged helix gene Mf1 is disrupted in the pleiotropic mouse mutation congenital hydrocephalus. Cell 93: 985–996. 9635428 10.1016/s0092-8674(00)81204-0

[7] Rice R, Rice DP, Olsen BR, Thesleff I (2003) Progression of calvarial bone development requires Foxc1 regulation of Msx2 and Alx4. Dev Biol 262: 75–87. 14512019 10.1016/s0012-1606(03)00355-5

[8] Mavrogiannis LA, Antonopoulou I, Baxova A, Kutilek S, Kim CA, et al (2001) Haploinsufficiency of the human homeobox gene ALX4 causes skull ossification defects. Nat Genet 27: 17–18. 11137991 10.1038/83703

[9] Jabs EW, Muller U, Li X, Ma L, Luo W, et al (1993) A mutation in the homeodomain of the human MSX2 gene in a family affected with autosomal dominant craniosynostosis. Cell 75: 443–450. 8106171 10.1016/0092-8674(93)90379-5

[10] Wuyts W, Reardon W, Preis S, Homfray T, Rasore-Quartino A, et al (2000) Identification of mutations in the MSX2 homeobox gene in families affected with foramina parietalia permagna. Hum Mol Genet 9: 1251–1255. 10767351 10.1093/hmg/9.8.1251

[11] Wilkie AO, Tang Z, Elanko N, Walsh S, Twigg SR, et al (2000) Functional haploinsufficiency of the human homeobox gene MSX2 causes defects in skull ossification. Nat Genet 24: 387–390. 10742103 10.1038/74224

[12] Satokata I, Ma L, Ohshima H, Bei M, Woo I, et al (2000) Msx2 deficiency in mice causes pleiotropic defects in bone growth and ectodermal organ formation. Nat Genet 24: 391–395. 10742104 10.1038/74231

[13] Berry FB, Saleem RA, Walter MA (2002) FOXC1 transcriptional regulation is mediated by N- and C-terminal activation domains and contains a phosphorylated transcriptional inhibitory domain. J Biol Chem 277: 10292–10297. 11782474 10.1074/jbc.M110266200

[14] Saleem RA, Banerjee-Basu S, Berry FB, Baxevanis AD, Walter MA (2001) Analyses of the effects that disease-causing missense mutations have on the structure and function of the winged-helix protein FOXC1. Am J Hum Genet 68: 627–641. 11179011 10.1086/318792 PMC1274476

[15] Wang X, Seed B (2003) A PCR primer bank for quantitative gene expression analysis. Nucleic Acids Res 31: e154. 14654707 10.1093/nar/gng154 PMC291882

[16] Berry FB, Skarie JM, Mirzayans F, Fortin Y, Hudson TJ, et al (2008) FOXC1 is required for cell viability and resistance to oxidative stress in the eye through the transcriptional regulation of FOXO1A. Hum Mol Genet 17: 490–505. 17993506 10.1093/hmg/ddm326

[17] Pierrou S, Hellqvist M, Samuelsson L, Enerback S, Carlsson P (1994) Cloning and characterization of seven human forkhead proteins: binding site specificity and DNA bending. Embo J 13: 5002–5012. 7957066 10.1002/j.1460-2075.1994.tb06827.x PMC395442

[18] Ichida F, Nishimura R, Hata K, Matsubara T, Ikeda F, et al (2004) Reciprocal roles of MSX2 in regulation of osteoblast and adipocyte differentiation. J Biol Chem 279: 34015–34022. 15175325 10.1074/jbc.M403621200

[19] Ittner LM, Wurdak H, Schwerdtfeger K, Kunz T, Ille F, et al (2005) Compound developmental eye disorders following inactivation of TGFbeta signaling in neural-crest stem cells. J Biol 4: 11. 16403239 10.1186/jbiol29 PMC1414066

[20] Seo S, Fujita H, Nakano A, Kang M, Duarte A, et al (2006) The forkhead transcription factors, Foxc1 and Foxc2, are required for arterial specification and lymphatic sprouting during vascular development. Dev Biol 294: 458–470. 16678147 10.1016/j.ydbio.2006.03.035

[21] Seo S, Kume T (2006) Forkhead transcription factors, Foxc1 and Foxc2, are required for the morphogenesis of the cardiac outflow tract. Dev Biol 296: 421–436. 16839542 10.1016/j.ydbio.2006.06.012

[22] Sommer P, Napier HR, Hogan BL, Kidson SH (2006) Identification of Tgf beta1i4 as a downstream target of Foxc1. Dev Growth Differ 48: 297–308. 16759280 10.1111/j.1440-169X.2006.00866.x

[23] Tamimi Y, Skarie JM, Footz T, Berry FB, Link BA, et al (2006) FGF19 is a target for FOXC1 regulation in ciliary body-derived cells. Hum Mol Genet 15: 3229–3240. 17000708 10.1093/hmg/ddl400

[24] Yamagishi H, Maeda J, Hu T, McAnally J, Conway SJ, et al (2003) Tbx1 is regulated by tissue-specific forkhead proteins through a common Sonic hedgehog-responsive enhancer. Genes Dev 17: 269–281. 12533514 10.1101/gad.1048903 PMC195981

[25] De Coster PJ, Mortier G, Marks LA, Martens LC (2007) Cranial suture biology and dental development: genetic and clinical perspectives. J Oral Pathol Med 36: 447–455. 17686002 10.1111/j.1600-0714.2007.00553.x

[26] Ishii M, Merrill AE, Chan YS, Gitelman I, Rice DP, et al (2003) Msx2 and Twist cooperatively control the development of the neural crest-derived skeletogenic mesenchyme of the murine skull vault. Development 130: 6131–6142. 14597577 10.1242/dev.00793

[27] Song HM, Fong KD, Nacamuli RP, Warren SM, Fang TD, et al (2004) Mechanisms of murine cranial suture patency mediated by a dominant negative transforming growth factor-beta receptor adenovirus. Plast Reconstr Surg 113: 1685–1697. 15114130 10.1097/01.prs.0000117363.43699.5b

[28] Hosokawa R, Urata M, Han J, Zehnaly A, Bringas P Jr, et al (2007) TGF-beta mediated Msx2 expression controls occipital somites-derived caudal region of skull development. Dev Biol 310: 140–153. 17727833 10.1016/j.ydbio.2007.07.038 PMC3337706

[29] Zarbalis K, Siegenthaler JA, Choe Y, May SR, Peterson AS, et al (2007) Cortical dysplasia and skull defects in mice with a Foxc1 allele reveal the role of meningeal differentiation in regulating cortical development. Proc Natl Acad Sci U S A 104: 14002–14007. 17715063 10.1073/pnas.0702618104 PMC1955817

[30] Rice DP, Rice R (2008) Locate, condense, differentiate, grow and confront: developmental mechanisms controlling intramembranous bone and suture formation and function. Front Oral Biol 12: 22–40. 18391493 10.1159/000115030

[31] Mears AJ, Jordan T, Mirzayans F, Dubois S, Kume T, et al (1998) Mutations of the forkhead/winged-helix gene, FKHL7, in patients with Axenfeld-Rieger anomaly. Am J Hum Genet 63: 1316–1328. 9792859 10.1086/302109 PMC1377542

[32] Nishimura DY, Swiderski RE, Alward WL, Searby CC, Patil SR, et al (1998) The forkhead transcription factor gene FKHL7 is responsible for glaucoma phenotypes which map to 6p25. Nat Genet 19: 140–147. 9620769 10.1038/493

[33] Bei M, Stowell S, Maas R (2004) Msx2 controls ameloblast terminal differentiation. Dev Dyn 231: 758–765. 15499554 10.1002/dvdy.20182

[34] Green PD, Hjalt TA, Kirk DE, Sutherland LB, Thomas BL, et al (2001) Antagonistic regulation of Dlx2 expression by PITX2 and Msx2: implications for tooth development. Gene Expr 9: 265–281. 11763998 10.3727/000000001783992515 PMC5964948

[35] Katagiri T, Yamaguchi A, Komaki M, Abe E, Takahashi N, et al (1994) Bone morphogenetic protein-2 converts the differentiation pathway of C2C12 myoblasts into the osteoblast lineage. J Cell Biol 127: 1755–1766. 7798324 10.1083/jcb.127.6.1755 PMC2120318

